# Vibrotactile Perception for Sensorimotor Augmentation: Perceptual Discrimination of Vibrotactile Stimuli Induced by Low-Cost Eccentric Rotating Mass Motors at Different Body Locations in Young, Middle-Aged, and Older Adults

**DOI:** 10.3389/fresc.2022.895036

**Published:** 2022-07-01

**Authors:** Ella Pomplun, Ashiya Thomas, Erin Corrigan, Valay A. Shah, Leigh A. Mrotek, Robert A. Scheidt

**Affiliations:** ^1^Department of Biomedical Engineering, Marquette University and Medical College of Wisconsin, Milwaukee, WI, United States; ^2^Department of Applied Physiology and Kinesiology, University of Florida, Gainesville, FL, United States

**Keywords:** sensory augmentation, acuity of vibration sensation, intensity discrimination, lifespan, aging

## Abstract

Sensory augmentation technologies are being developed to convey useful supplemental sensory cues to people in comfortable, unobtrusive ways for the purpose of improving the ongoing control of volitional movement. Low-cost vibration motors are strong contenders for providing supplemental cues intended to enhance or augment closed-loop feedback control of limb movements in patients with proprioceptive deficits, but who still retain the ability to generate movement. However, it remains unclear what form such cues should take and where on the body they may be applied to enhance the perception-cognition-action cycle implicit in closed-loop feedback control. As a step toward addressing this knowledge gap, we used low-cost, wearable technology to examine the perceptual acuity of vibrotactile stimulus intensity discrimination at several candidate sites on the body in a sample of participants spanning a wide age range. We also sought to determine the extent to which the acuity of vibrotactile discrimination can improve over several days of discrimination training. Healthy adults performed a series of 2-alternative forced choice experiments that quantified capability to perceive small differences in the intensity of stimuli provided by low-cost eccentric rotating mass vibration motors fixed at various body locations. In one set of experiments, we found that the acuity of intensity discrimination was poorer in older participants than in middle-aged and younger participants, and that stimuli applied to the torso were systematically harder to discriminate than stimuli applied to the forearm, knee, or shoulders, which all had similar acuities. In another set of experiments, we found that older adults could improve intensity discrimination over the course of 3 days of practice on that task such that their final performance did not differ significantly from that of younger adults. These findings may be useful for future development of wearable technologies intended to improve the control of movements through the application of supplemental vibrotactile cues.

## Introduction

Proprioceptive kinesthesia, the sense of body posture and movement (1), is critical to independent living because sensory feedback of body configuration is required for the accurate planning and control of movement. An important source of proprioceptive kinesthesia derives from mechanoreceptors in the muscles, joints, and skin of the limbs and trunk (1). These sources of proprioceptive sensation are an important component of afferent information that the central nervous system (CNS) uses to complete even the simplest tasks, like reaching to pick up a cell phone (2). Feedback pathways serving proprioceptive kinesthesia can be degraded in diseases such as Parkinson's (3), multiple sclerosis (4), or stroke (5). This leads to unsteady or poorly controlled movements. When somatosensory contributions to proprioceptive kinesthesia are compromised, patients can often rely on vision to help control their limbs, but long processing delays inherent to the visual system [100–200 ms; (6)] yield movements that are typically slow, poorly coordinated, and require great concentration (7, 8). Visually guided corrections come too late and result in jerky, unstable movements (9). The long term goal of our work is to improve motor function in individuals with impaired proprioceptive sensation of limb posture and movement by using sensory augmentation to circumvent impaired kinesthetic feedback pathways and recruit new pathways involving the tactile stimulation of different - and potentially non-moving - body parts [cf. (10)].

Non-invasive body-machine interfaces are being developed to mitigate a number of sensorimotor impairments due to disease and injury [c.f., (11–16)]. To advance our long-term goal, we specifically seek to develop and use low-cost technology to enhance or augment closed-loop feedback control of limb movements in patients with proprioceptive deficits, but who still retain the ability to generate movement. Sensory interfaces using auditory, haptic, electrical, or vibrotactile stimulation have been proposed to mitigate a variety of other sensory impairments [see (17) for a review]. Of these stimulation technologies, vibrotactile feedback (VTF) appears particularly well-suited to convey supplemental movement information without taxing users' auditory or visual attention, and without interfering with important functions like speaking and self-feeding. VTF also has the practical benefit of being non-invasive and inexpensive to implement.

There are many ways to encode information relevant to limb movement control into vibrotactile signals for sensory augmentation. Four categorically different methods of temporal encoding of information include: continuous state feedback relative to an arbitrary reference body configuration (18, 19); continuous error feedback relative to a goal (11, 19–24); continuous optimal feedback relative to some (arbitrary) cost function (25); and intermittent indication of undesirable conditions [i.e., alarms; cf., (26)]. Spatially distributed stimulation at different locations can also be used to convey symbolic information (27, 28). Regardless of which form VTF takes, the cues must be designed and applied such that the encoded information can be easily perceived and interpreted by the user [cf., (29–31)]. How should vibrotactile feedback be designed to ensure perceptibility of movement-related information? The immediate goal of the present study is to advance understanding of vibrotactile perception using low-cost wearable technology ultimately expected to deliver information about movement kinematics non-invasively to survivors of neural injury.

Several recent studies have shown that low-cost, eccentric rotating mass (ERM) motors can be used to deliver time-varying, suprathreshold VTF to multiple locations on the arm (18, 19, 32) and hand (24), thereby enhancing the real-time control of reaching movements of the other arm in healthy people and in stroke survivors [c.f., (10, 25)]. In those studies, real-time hand position was encoded in VTF signals provided by up to two simultaneous time-varying vibrotactile stimuli presented at different locations on one arm; the participant's task was to use the information encoded in those signals to help them move and stabilize the other arm and hand. While stimulation sites on the non-moving arm and hand proved viable for many participants, a few struggled to discriminate between the applied stimuli, suggesting that the upper extremity may not be an ideal site for all users. Although prior research has found differences in vibrotactile frequency discrimination in glabrous vs. hairy skin (33), vibrotactile discrimination thresholds at locations beyond the upper extremity have rarely been studied. This study seeks to address that knowledge gap.

This translational study contributes to a line of research that uses low-cost devices to provide vibrotactile feedback as a practical method of sensory augmentation for people with brain injuries leading to the loss of proprioceptive kinesthesia arising from mechanoreceptors in certain parts of the body [cf., (10)]. For the system to have utility, users must be able to discriminate the time-varying intensity of the vibrotactile stimuli to infer the hand's location in space, and then use that information to guide action [cf., (19)]. Recognizing that different injuries can give rise to somatosensory deficits localized to just one side of the body or to only some limbs (34) and that the severity of injury is a main factor influencing somatosensory impairment [cf., (35)], the ideal location for VTF application may vary from individual to individual. Moreover, the incidence of stroke increases with age (36); any new technology intended to improve quality of movement and quality of life after stroke should be usable by people of all ages, including older adults who often exhibit decreases in sensory sensitivity with increasing age (37–39) and acuity (40). Can such age-related perceptual deficits be overcome with sensory discrimination training?

This study sought to identify and compare the acuity of vibrotactile stimulus intensity discrimination (i.e., the just noticeable difference JND between stimuli of different intensities) at various possible stimulation sites across the body using low-cost vibration motor technology appropriate for use as stimulators in sensory augmentation applications. We also sought to determine the extent to which the capability to discriminate between stimuli delivered by low-cost stimulators varies as a function of age, and if so, whether age-related effects can be mitigated through perceptual training on vibrotactile stimulus intensity discrimination. As an early step toward our long term goal, we focus on vibrotactile perception in neurologically-intact individuals spanning a wide age range. As such, the study builds on and extends a long history of research examining the impact of stimulation site and aging on vibrotactile *detection* thresholds [i.e., the smallest perceptible stimulus; cf., (37, 41–43)]. Given that vibrotactile detection thresholds vary across body locations (37, 43), we expect also to find differences in vibrotactile stimulus intensity *discrimination* across different stimulation sites. Further, as many aspects of sensory perception commonly decrease even in healthy older adults (37–40), we expect to find differences in vibrotactile stimulus intensity discrimination across the lifespan.

## Materials and Methods

### Participants

Healthy adults, aged 18 to 89 years, provided written informed consent to participate in experiments that assessed their capability to perceive differences in the intensity of brief vibrotactile stimuli applied at various body locations. None of the participants had known motor, tactile, or cognitive deficits. The first set of experiments used low-cost eccentric rotating mass vibration motors to quantify and compare the acuity of vibrotactile perception across four different body locations. These single-session experiments involved 32 participants (18 female; 14 male). A “younger adults” group (18–39 years) comprised sixteen individuals (23.75 ± 4.1 years; mean ± 1 SD). A “middle age” group (40–60 years) comprised six individuals (53.5 ± 5.9 years). An “older adults” group (61+ years) comprised ten individuals (74.7 ± 7.1 years). Sixteen participants (three young, five middle, eight older) had prior experience with vibrotactile discrimination testing (<3 h each). In another set of experiments, sixty-five naïve participants (39 female; 26 male) engaged in three testing sessions (<1 h each) designed to measure the effect of age and practice on their capability to perceive differences in vibrotactile stimulation on the forearm. The three sessions were performed on separate days spaced >24 h apart. Here again, participants were divided into three age groups for subsequent analyses: younger adults (23.8 ± 5.47 years, *n* = 12), middle-aged adults (53.5 ± 5.54 years, *n* = 19), and older adults (75.6 ± 6.86 years, *n* = 34). All experimental procedures were approved by the Institutional Review Board at Marquette University in full accordance with the Declaration of Helsinki.

### General Experimental Setup

**Figure 1:**
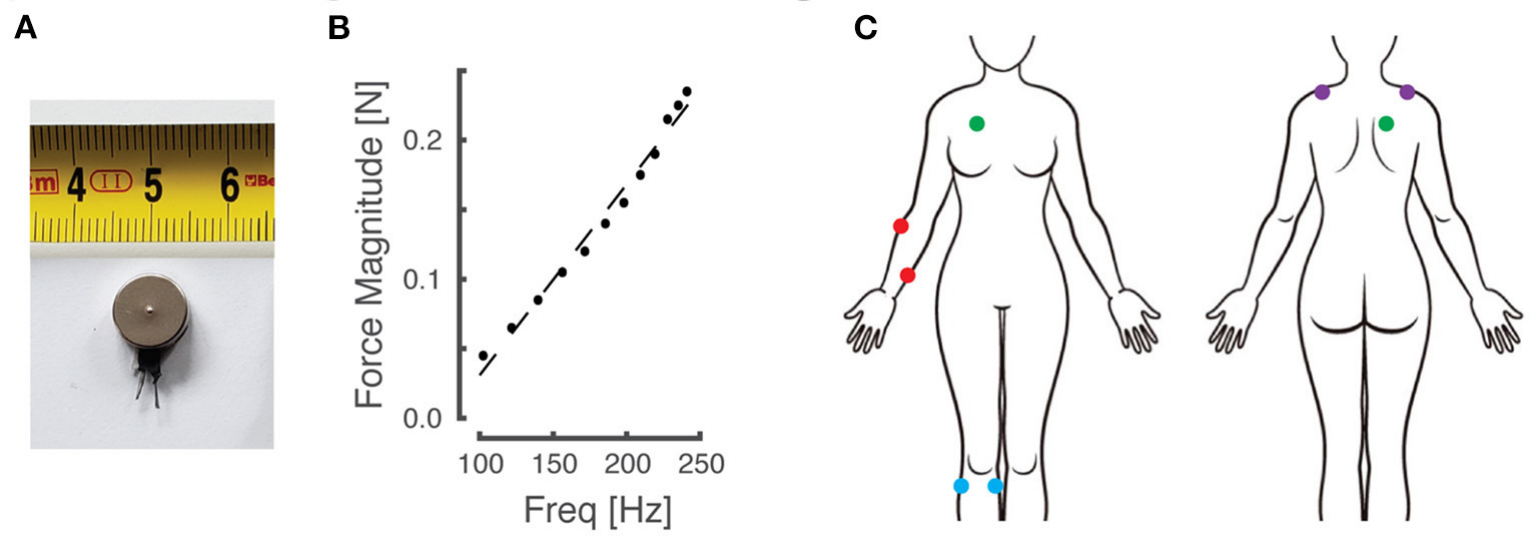
Materials and experimental setup. **(A)** An example of the eccentric rotating mass (ERM) vibration motors used for this study. **(B)** Empirical relationship between vibration frequency and magnitude, which co-vary monotonically as a function of motor activation in the ERM motors used in this study. **(C)** Schematic of the body with vibration motor locations indicated by colored dots. Red: right forearm; purple: right/left shoulders; blue: right knee; green: right torso.

Participants sat in a comfortable high-backed chair while engaging in the experiments, which involved several sets of 2-alternative forced choice (2AFC) testing blocks. Depending on the experiment (described below), a pair of miniature, low-cost, eccentric rotating mass (ERM) vibration motors (Precision Microdrives Ltd, Model #310-117; Figure 1A) was affixed to the body at one of four different paired locations using 4 cm strips of Transpore tape (3M Inc). The vibration motors have a specified operational frequency range of 60–240 Hz and a vibration amplitude (applied force) ranging from approximately 0.02 N to 0.24 N. Vibration amplitude increases monotonically as a function of vibration frequency over the range of motor activation values for the ERM motors used in this study [Figure 1B; see also Table A1 in (44)]. The vibration motors were powered by drive circuitry that controlled their activation through pulse width modulation (45). The drive circuitry was interfaced to a laptop computer running a control algorithm scripted within the MATLAB computing environment (version R2017a; the MathWorks Inc., Natick MA).

Although vibration frequency covaries with amplitude in the ERM vibration motors used in this study (Figure 1B), we chose to report vibrotactile stimulus intensity only in terms of frequency for narrative simplicity. The fact that stimulus frequency and amplitude are coupled in these low-cost vibration motors is not a limitation of our approach for two reasons. First, experimental evidence shows that people can discriminate vibrotactile stimuli better when amplitude and frequency change coherently [e.g., both increasing or both decreasing together; cf., (44)]. Second, our translational study sought to compare perceptual acuity across body locations, age groups, and repeated bouts of practice using practical, low-cost stimulators suitable for wearable sensory augmentation.

**Figure 2:**
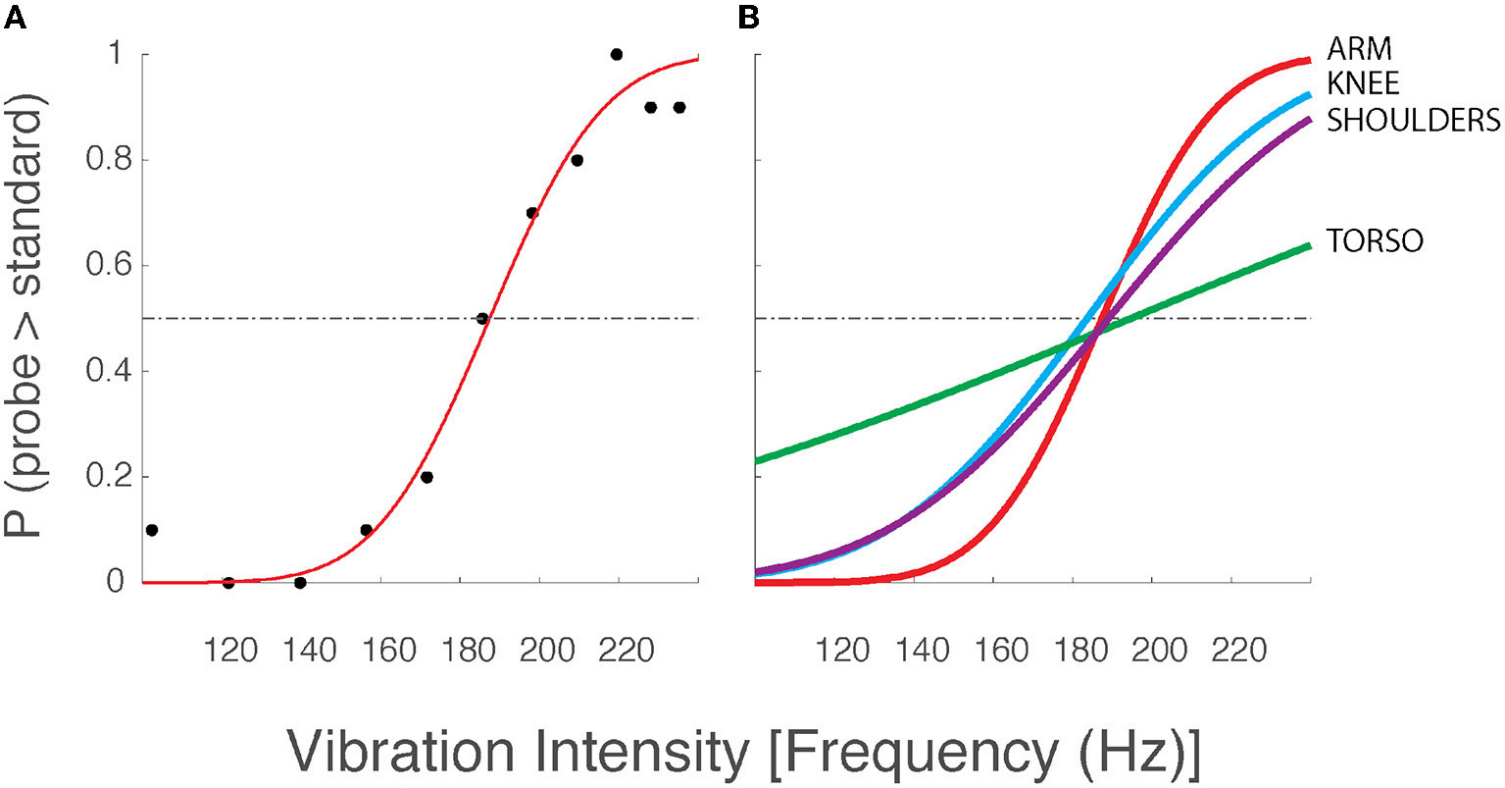
Two-alternative forced choice data and best-fit psychometric curves from a selected participant from Experiment 1. **(A)** Data from a single testing block with vibration motors applied to the arm. Although vibration frequency covaries with amplitude in the ERM vibration motors used in this study (see Figure 1B), we chose to report vibrotactile stimulus intensity only in terms of frequency for narrative simplicity. Black dots: the observed intensity at which the participant responded that the corresponding probe stimulus intensity was greater than the standard stimulus (186 Hz). Red line: the best fit psychometric curve fit to the observed experimental data. Horizontal dashed line: chance probability. **(B)** Best fit psychometric curves from the same participant at the arm (red), knee (blue), shoulders (purple), and torso (green).

Each 2AFC testing block took about 5 min to complete. These experiments used the method of constant stimuli (46) to determine the just noticeable difference (JND) between vibrotactile stimuli of different intensities. Under the 2AFC protocol, participants were presented with 110 paired vibrotactile cues. Each cue included a variable probe stimulus and a standard stimulus that remained constant across each trial. The standard stimulus' intensity corresponded to a frequency (186 Hz) that is well-within the Pacinian Corpuscle's frequency sensitivity band [which ranges from approximately 40 Hz to at least 400 Hz; see (42, 47, 48), and (49); but see also (50)]. The probe stimulus could take on one of 11 different values ranging from 100 Hz to 235 Hz: five frequency values were distributed above the standard stimulus, five values were distributed below the standard, and one stimulus value was equal to that of the standard (cf., black symbols in Figure 2, left panel). This range of probe frequencies spanned the specified operating activation range of the low-cost vibration motors. Each probe frequency was presented 10 times per experiment.

In some testing blocks, the vibrotactile stimuli were presented simultaneously, i.e., both motors vibrated at the same time for 750 ms. In other blocks, stimuli were presented sequentially. In this condition, one of the vibrotactile stimuli (standard or probe; pseudorandomly selected) was presented through one motor for 750 ms. This step was followed by a 750 ms pause, whereupon the other stimulus was presented through the same motor or the other motor for 750 ms as described in greater detail below. This stimulus timing was chosen to balance a tradeoff between providing participants with enough time to form a stable percept within each stimulus interval, vs. the overall duration of the entire experimental session [see also (42)]. After both stimuli were presented (either simultaneously or sequentially), participants indicated verbally and unambiguously which of the two stimuli they perceived to be greater in vibrotactile intensity (e.g., “first” or “second;” “left” or “right;” “front” or “back”). Responses were keyed into the data collection computer by the experimenter before presentation of the next stimulus pair. To mask potential auditory cues about vibration intensity, participants wore closed-back, on-ear headphones (Beats Solo3; Apple, Inc.) that played white noise stimuli throughout the duration of each experiment.

### Experiment 1 - Effects of Stimulus Location on Vibrotactile Discrimination

In this set of experiments, participants engaged in four sets of two, 2-alternative forced choice (2AFC) experiments (a total of eight testing blocks per participant). Prior to each set of two 2AFC blocks, the vibration motors were placed on one of four different paired body locations (Figure 1C). Locations included: the forearm on dermatomes C7 and T1 (on the skin over the extensor carpi radialis and flexor digitorum superficialis muscle bodies), the knee on dermatomes L3 and L5 (over the medial gastrocnemius and tibialis anterior muscles), the anterior-posterior (AP) torso on dermatomes C4 and T2 (over the pectoralis major and rhomboid major muscles), and on the shoulders at dermatome C4 (over the left and right superior fibers of the trapezius muscle). Note that by applying vibration over the muscle bellies (rather than to their tendons) and by vibrating at frequencies greater than 80 Hz, contributions of muscle spindle afferents to the perceptual response should be strongly limited [cf., (51)]. In all cases, the stimulation sites were chosen such that the vibrators were spaced at least 6 cm apart to minimize the potential for mechanical cross-talk between stimulation sites [cf., (45)]. Apart from the shoulder condition, all vibrator placements were exclusively on the participant's right side, regardless of hand dominance. The sequence of tested locations was randomized across participants. In one of the testing blocks performed at each body location, the vibrotactile stimuli were presented simultaneously, i.e., both motors presented vibrotactile stimuli at the same time. In the other testing block, stimuli were presented sequentially. The order of stimulus presentation conditions {sequential, simultaneous} was also randomized at each stimulation site for each participant.

### Experiment 2 - Effects of Practice on Vibrotactile Discrimination

In this set of experiments, participants engaged in a series of four, 2-alternative, forced choice (2AFC) experiments that were repeated in three testing sessions that were each spaced more than 24 h apart. On each day, the participants sat next to a table with their right arm resting comfortably on a soft cushion on the table's surface with the shoulder flexed approximately 10° and the elbow was flexed approximately 30°. A pair of vibration motors was affixed to the participant's right forearm with one placed on the skin of dermatome C7 over the extensor carpi radialis muscle, while the other was placed on dermatome T1 on the medial forearm and at least 8 cm from the C7 stimulation site (Figure 1C). Depending on the test block, the two vibrators could either vibrate simultaneously at both locations (C7T1SIM), sequentially across dermatomes (the C7T1SEQ condition), or sequentially at only one of the locations (i.e., the within-dermatome C7SEQ and T1SEQ conditions). In the C7T1SEQ testing block, the C7 vibrator always vibrated first followed by the T1 vibrator; the presentation order of probe and standard stimuli were randomized across the 110 paired stimulus presentations within each test block. After each presentation, the participants verbally indicated which stimulus they perceived as more intense. Once their response was recorded, the next pair of stimuli was generated after a brief pause. The order of the four testing conditions was counterbalanced across participants and across testing days.

### Data Analysis

Participant responses were converted into probabilities of perceiving the probe stimulus as greater in intensity than the standard stimulus. A psychometric function was fit to the probability data from each participant and testing block (110 assessments each) as a function of probe stimulus frequency using a cumulative normal distribution:


(1) $$\begin{array}{l} F\left(x\right)=\frac{1}{2}\left[1+erf\left(\frac{x-\mu }{\sigma \sqrt{2}}\right)\right]. \end{array}$$

F(x) is the predicted probability, x is the probe frequency, μ is the mean of the underlying decision process modeled as a normal distribution, σ is the standard deviation of that normal distribution, and *erf* is the cumulative normal function. The MATLAB function fminsearch was used to find values of μ and σ that minimize the sum of square error between the model-predicted and actual probabilities. The discrimination threshold (i.e. JND) for each participant was defined as one standard deviation of the underlying normal distribution, i.e. the σ found by fminsearch.

### Statistical Hypothesis Testing

Several experimental studies have found that the *detection* sensitivity to vibrotactile stimuli varies across the body (37, 43); here we tested how sequential and simultaneous *discrimination* thresholds for ERM vibrotactile stimuli might vary across body locations. It is also known that sensory sensitivity (37–39) and acuity (40) decrease with aging; we therefore tested how sequential and simultaneous vibration intensity discrimination thresholds for ERM stimuli might vary across the lifespan.

All statistical testing was done with IBM SPSS software (version 25). Prior to running parametric tests, we inspected the discrimination threshold data for skewness, kurtosis, and normality. Due to its power with smaller sample sizes, we tested for normality using the Shapiro-Wilk test. This test revealed that the discrimination threshold data departed significantly from normality (*p* < 0.0005) with respect to all independent variables (age, location, presentation timing). Skewness ranged from 1.34 to 1.65 and kurtosis ranged from 2.62 to 3.24. Thus, to perform parametric tests, the data were transformed using the Rank Transform, Inverse Normal Transform (RT-INT) method as described by Lupsen (52), which ensures that the transformed data are normally distributed. Briefly, according to this method, the discrimination threshold data were pooled across the entire study, each threshold observation was ranked, and then normal scores were computed as per Equation 2:


(2) $$\begin{array}{l} RTINT=\;\phi^{-1}\left(\frac{R_{i}}{n+1}\right) \end{array}$$

where ϕ^−1^ is the inverse normal transform, *R*_*i*_ is the rank of the *i*^*th*^ observation, and *n* is the number of observations. From there, the transformed data were used as the dependent variable to perform separate mixed model ANOVA and *post-hoc* paired samples *t*-tests to compare vibrotactile discrimination thresholds between age groups, and between presentation timing conditions across different body locations (Experiment 1) and across training sessions (Experiment 2). Statistical significance was set at a family-wise error rate of α = 0.05.

## Results

Participants performed 2-alternative, forced choice (2AFC) experiments that assessed vibrotactile stimulus intensity discrimination thresholds at four different body locations (shoulders, AP torso, forearm, knee) in a single experimental session (Experiment 1) and at just the forearm in a series of experiments performed on separate days (Experiment 2). In each experiment, eccentric rotating mass (ERM) motors presented a pair of brief vibrotactile stimuli either simultaneously or sequentially. Each pair of stimuli included a “standard” stimulus at a constant frequency and a probe stimulus, which could take on one of eleven different values. Participants were to indicate which stimulus they perceived to be more intense. All participants indicated that they understood the task instructions prior to experimental testing, and all participants appeared attentive throughout their testing session(s). None of the subjects mentioned perceiving any illusory motion in the stimulated arm at any time during testing.

### Experiment 1 - Effects of Stimulus Location on Vibrotactile Discrimination

Figure 2A shows the logistic curve (Equation 1) fit to the response data from a single selected testing condition from a single participant in the older adult group of Experiment 1. The estimated likelihood that the participant verbally indicated the probe stimulus to be more intense than the standard stimulus is presented for all eleven probe frequencies (black dots). Below the standard stimulus intensity (186 Hz), we expect the probability to be low (<0.5), whereas we expect higher choice probabilities at intensities above the standard stimulus intensity. The results conformed to these expectations in nearly every case for every participant. For the experimental data shown in Figure 2, the best-fit curve is flat and near zero at the lowest probe intensities indicating the participant had a near-perfect capability to perceive low-intensity probe stimuli as being less intense than the standard stimulus. As the probe intensity approached that of the standard, the value of the best-fit curve approached 0.5 indicating a near-chance probability of declaring the probe stimulus greater than the standard. At higher probe intensities, the line asymptotes at values near 1.0, indicating that the participant had a high probability of indicating the probe stimulus to be more intense than the standard. The slope of the best-fit curve as it passes through the value *p* = 0.5 corresponds to the precision with which the participant was able to discriminate probe stimuli from the standard. This slope is determined by the σ value of the underlying normal function used to model the decision process. We operationally defined the vibrotactile discrimination threshold (or just noticeable difference, JND) to be equal to 1 σ of the underlying normal function. Curves with steeper slopes indicate lower discrimination thresholds for the given condition, i.e., better perceptual acuity.

Figure 2B shows the best-fit curves for stimuli applied to all four body locations for the same selected participant whose data were shown in Figure 2A. Here, the curves indicate that the participant's vibration intensity discrimination acuity was better for simulation sites on the arm, knee, and shoulders than on the AP torso. Similar results were found across the study cohort. Across participants, average thresholds varied across stimulation sites such that the difference limen (Δ F/F; i.e., JND/standard) at the shoulders (24.2%) was less than that across the torso (33.3%).

**Figure 3:**
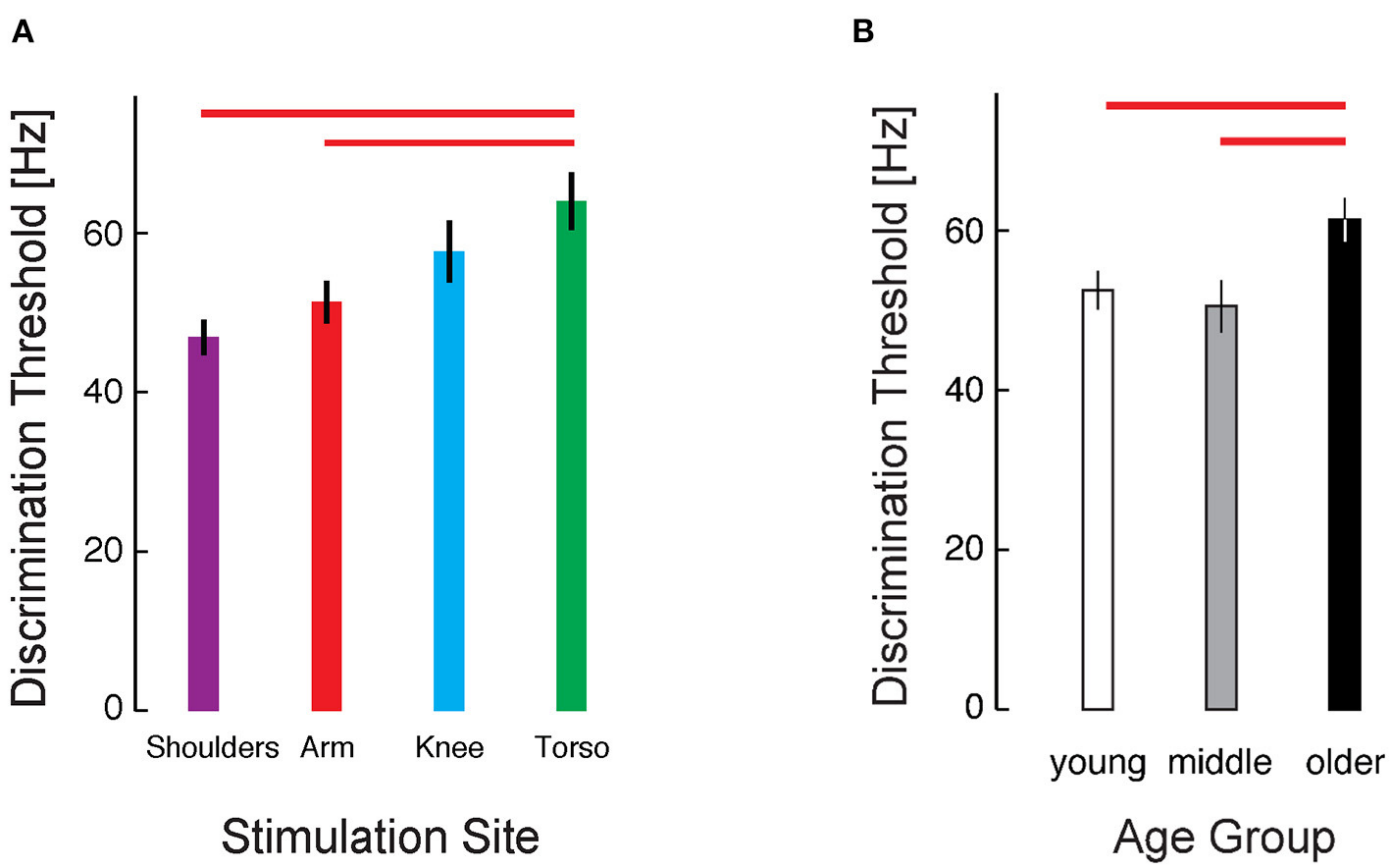
Cohort results from Experiment 1. **(A)** Average vibrotactile discrimination thresholds (mean ± 1 SEM) at each stimulus location for all participants. Red bars indicate significant differences between the arm and torso (*p* = 0.034) and the shoulder and torso (*p* = 0.003) identified after RT-INT transformation and subsequent statistical analysis. Participants performed worse (i.e., they had higher vibrotactile thresholds) when the vibratory stimuli were applied across the torso vs. when they were applied at the other locations. **(B)** Vibrotactile discrimination thresholds (mean ± 1 SEM) averaged across experimental conditions within each age group. Red bars indicate significant differences between the older adult age group and the middle-age (*p* = 0.015) and young adult (*p* = 0.008) groups. Note again that we report vibrotactile stimulus intensity in terms of frequency for narrative simplicity even though vibration amplitude covaries with frequency in the ERM vibration motors used in this study.

To explore the cohort data, we applied a three-way mixed model ANOVA to the RT-INT transformed threshold data obtained in Experiment 1, with stimulus location, presentation timing, and age group as independent fixed factors. We identified a main effect of stimulus location on vibrotactile intensity discrimination (ANOVA: F_3, 224_ = 4.457, *p* = 0.005). *Post-hoc* testing revealed significant differences between locations, with the raw (untransformed) torso thresholds 25% greater than at the arm (Cohen's d = 0.65, a moderate effect size), and the torso thresholds 36% greater than at the shoulders (d = 0.96, a larger effect size) (Figure 3A).

We also identified a main effect of age group on the transformed threshold data (F_2, 224_ = 6.282, *p* = 0.002). *Post-hoc* testing revealed a 17% difference in thresholds between the young and older age groups (d = 0.69) and a 21% difference between the middle and older age groups (d = 0.77) (Figure 3B). No significant differences were found between the young and middle age groups.

All participants performed two test blocks at each location: one block with the stimuli presented simultaneously and the other block with the stimuli presented sequentially. Although measured intensity discrimination thresholds tended to be lower (and acuity higher) for sequentially-presented stimuli than for simultaneously-presented stimuli (sequential: 51.28 Hz ± 21.77; simultaneous: 59.48 Hz ± 28.85), this difference did not quite reach statistical significance after RT-INT transformation (F_1, 224_ = 2.92, *p* = 0.089). The results of ANOVA also did not identify any significant two- or three-way interactions between factors (*p* > 0.21 in each case).

We repeated our analysis of variance to address the fact that a subset of the participants in Experiment 1 were not completely naïve to the vibrotactile intensity discrimination task; we included as a covariate a Boolean variable indicating prior experience with stimulus intensity discrimination. The results obtained were virtually identical to those reported above. As such, the results reported in Experiment 1 were not meaningfully impacted by brief prior experience with the vibrotactile intensity discrimination task.

**Figure 4:**
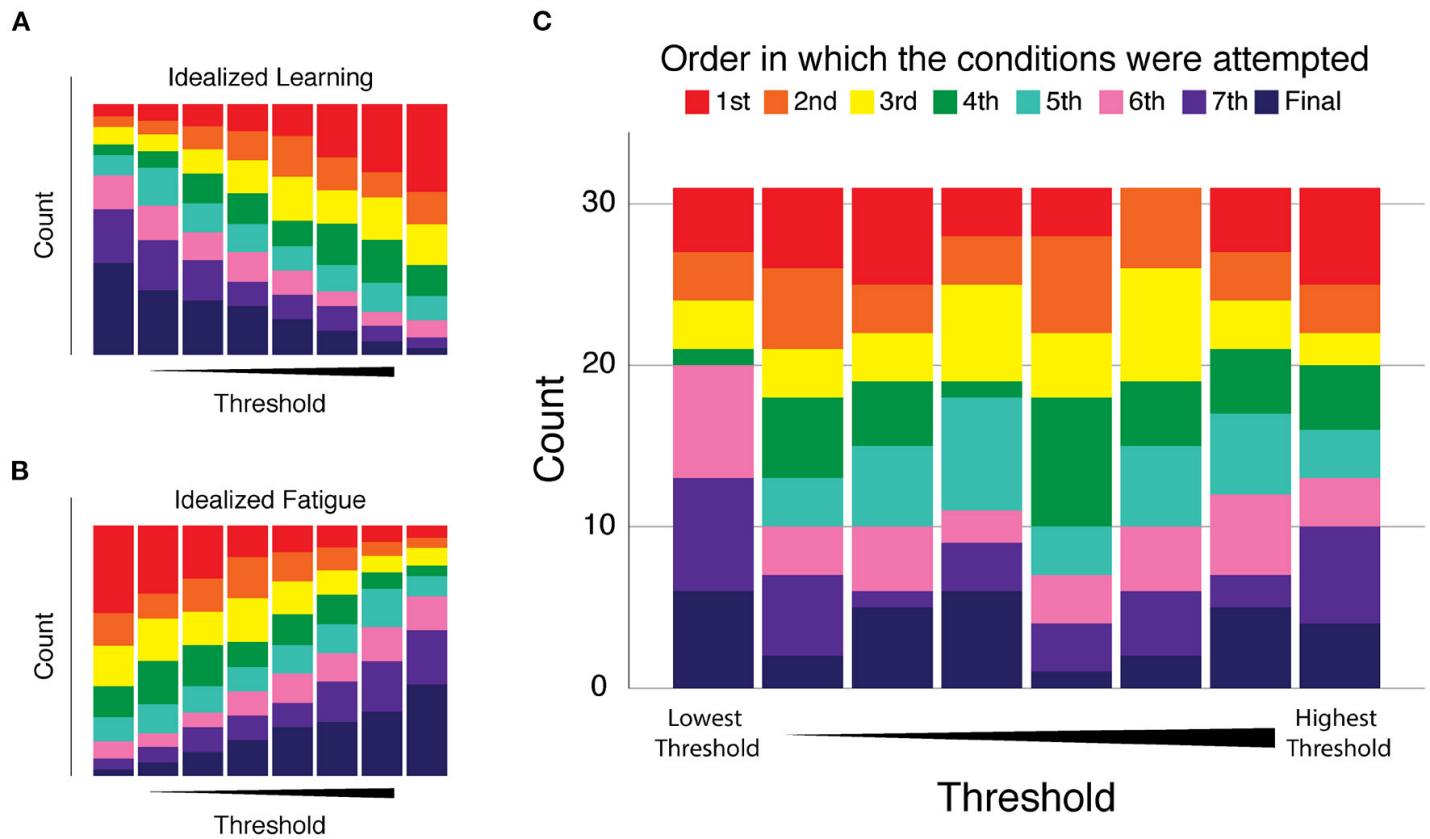
Analysis of performance changes over the course of the eight 2AFC experiments of Experiment 1. Condition Order: the order in which the conditions were performed were numbered and color coded from one (red) to eight (dark blue). Threshold Order: the ranked magnitude of each participant's discrimination thresholds from smallest (best) to largest (worst). Each plot presents the number of times (count) each condition resulted in the smallest threshold (left-most stacked bar) on up to the largest threshold (right-most stacked bar). **(A)** Idealized “learning” condition modeled as a consistent trend of improvement with practice as the experiments progress. **(B)** Idealized “fatigue” modeled as a consistent trend of decreasing performance as the experiments progressed. **(C)** Cohort results: we observed no consistent trend of performance change as a function of condition order. The disorganized pattern of bar sizes of all colors indicates that smaller and larger thresholds were equally likely to occur at any time during the experimental session.

Finally, we assessed whether participants might have exhibited systematic changes in performance over the course of their eight 2AFC test blocks. Such changes could reflect an improvement over the course of the single session experiment due to practice effects (i.e., learning), or it could reflect a decrement in performance due to fatigue. In response to these possibilities, we determined for each participant the order in which the conditions were performed and numbered them from one to eight, referring to this arrangement as *Condition Order*. For each participant's thresholds we ranked them from one (being the best/smallest) to eight (being the largest) and we called this variable *Threshold Order*. We counted the number of times the first condition resulted in the smallest threshold and then the second condition and so on. The results of this analysis are presented in Figure 4. If there was a consistent trend of improvement with practice during the experiments, we would expect that the smallest thresholds would occur more often in conditions performed later, i.e., that Figure 4 would have large purple and pink bars on the left side of the graph and the right side of the graph would have large blue and red bars (see the graphical example shown in Figure 4A). Alternatively, if performance decreased as the experiments progressed as in a fatigue situation, the largest thresholds would occur later in the session; there would be large bars of pink and purple on the right side of the graph and large blue and red bars on the left side of the graph (see Figure 4B). Across all participants, neither of these situations occurred. Figure 4C shows that the smaller and larger thresholds were equally likely to occur at any time during the experimental session, and thus differences in vibrotactile discrimination threshold are more strongly related to stimulus location and participant age than testing condition order in this experiment.

### Experiment 2 - Effects of Practice on Vibrotactile Discrimination

In this experiment, we specifically investigated the influence of age and practice on the capability to discriminate between two vibrotactile stimuli presented sequentially and simultaneously within and across dermatomes. We hypothesized that the acuity of vibration sensation would differ across age group, condition, and day. To test this, we applied mixed model ANOVA to the RT-INT transformed threshold data obtained in Experiment 2 to assess potential main and interaction effects between the independent factors: testing condition, age group, and testing session (i.e., day).

**Figure 5:**
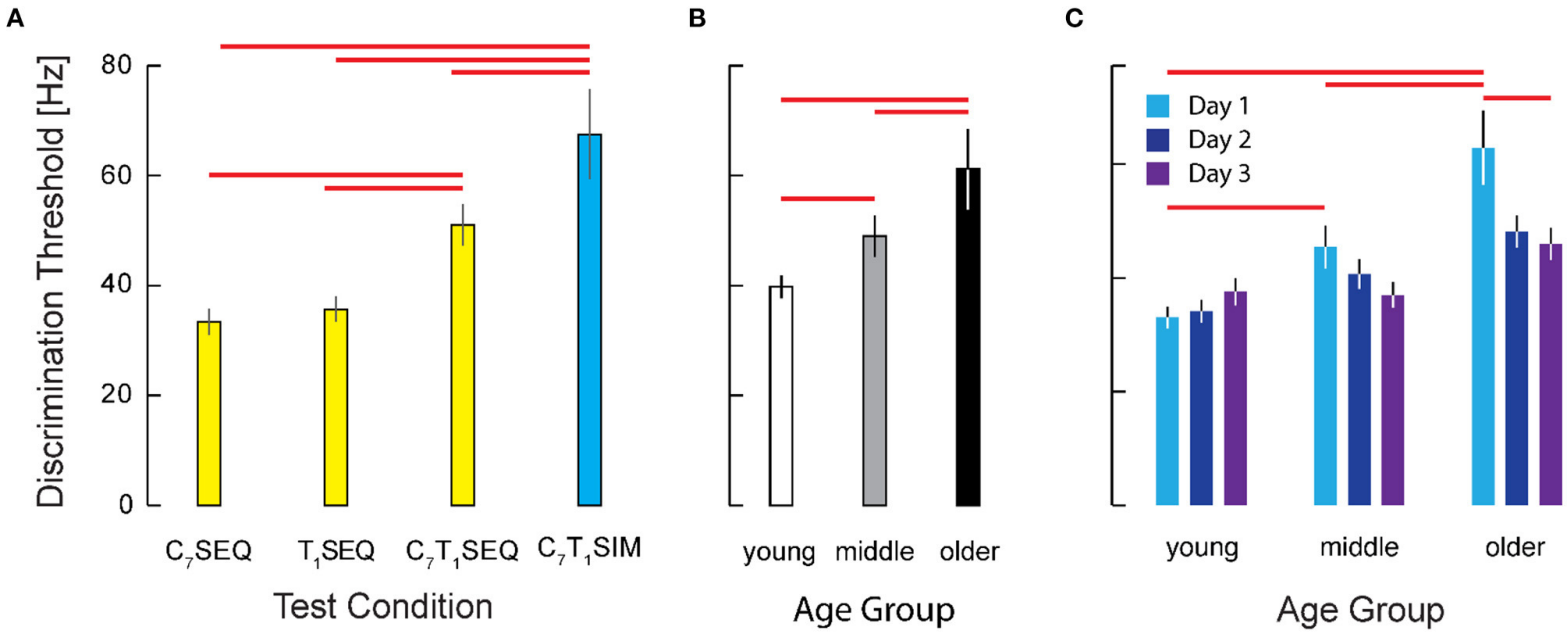
Cohort results from experiment 2. **(A)** Vibrotactile discrimination threshold as a function of testing condition. Participant threshold data from the two within-dermatome conditions (C7SEQ and T1SEQ) exhibited greater discrimination acuity that the two between-dermatome conditions. Note also that the acuity of the condition with simultaneous stimulation was significantly worse than the acuities observed in all three of the sequential testing conditions. Error bars: ± 1 SEM. Red horizontal significance bars: *p* < 0.05 after correction for multiple comparisons. **(B)** Main effect of age on Vibrotactile discrimination threshold. **(C)** Interaction between age group and testing day. Note the significant training effect across days in the older age group (but not the younger or middle-age groups).

As determined by ANOVA, we identified a main effect of testing condition {C7T1SIM, C7T1SEQ, C7SEQ, T1SEQ} on the transformed threshold data (F_(3, 626)_ = 87.74; *p* < 0.0005) (Figure 5A). *Post-hoc* testing of participant performance found significant differences between the intensity discrimination thresholds in the four experimental conditions; both of the within-dermatome sequential testing conditions C7SEQ (33.4 Hz) and T1SEQ (35.7 Hz) exhibited lower thresholds (i.e., higher acuity) than both between-dermatome conditions (C7T1SEQ: 51.0 Hz; C7T1SIM: 67.4 Hz). *Post-hoc* testing also found significantly higher vibrotactile intensity discrimination threshold (lower acuity) in the C7T1SIM condition than in the C7T1SEQ condition. Performance in the two within-dermatome conditions (C7SEQ and T1SEQ) did not different from each other. The effect size of the C7T1SEQ vs. C7SEQ and C7T1SIM vs. C7SEQ contrasts were both large (d = 1.33 and d = 1.70, respectively).

We also identified a main effect of age group {younger, middle-aged, older} on the transformed threshold data (F_(2, 626)_=33.15, *p* = 0.0005). *Post-hoc* testing showed significant differences in vibrotactile stimulus intensity discrimination between participants in the older group (61.2 Hz) and both the young (39.8 Hz) and the middle (49.0 Hz) age groups (*p* < 0.0005 in both cases; see Figure 5B). By contrast, we found no significant difference between vibrotactile thresholds in the young and middle age groups. The effect sizes for the contrasts between the older adults and the younger and middle-aged participants were d = 0.96 and d = 0.64, respectively.

We also hypothesized that participants might improve their tactile discrimination ability with practice. Although we observed no support for a main effect of testing day on the acuity of vibrotactile discrimination (Figure 5C), the results of ANOVA did indicate a significant interaction between day and age group (F_(4, 626)_= 2.84, *p* = 0.024) such that the older adults exhibited substantial improvement over the 3 days of testing whereas the two younger groups were at least as good on the first day as they were on the third testing day (Figure 5D). *Post-hoc* testing revealed no significant difference across days for the young and middle age groups, whereas the older participants did show a significant improvement from Day 1 to Day 3 (*p* = 0.003). For the older participants in particular, 2 days of practice had a medium-sized effect on vibrotactile intensity discrimination (d = 0.47). Whereas on Day 1 the older adults performed worse than both the younger and middle aged groups (*p* = 0.001 and *p* = 0.046, respectively), by Day 3, the older participants had improved such that their performances did not differ significantly from those of the younger and middle-aged groups (*p* > 0.230 in both cases).

## Discussion

We performed two sets of experiments that characterized the acuity of vibrotactile stimulus intensity discrimination at various sites on the body. In one set of experiments, healthy human participants performed a series of 2-alternative, forced choice experiments that quantified their capability to perceive small differences in the intensity of stimuli provided by low-cost eccentric rotating mass vibration motors affixed to the arm, leg, shoulders, and AP torso. Consistent with prior studies of vibrotactile stimulus *detection* (37, 43), we found systematic differences in the acuity of stimulus intensity *discrimination* across locations such that discrimination was better for stimuli presented to the arm and shoulders than for stimuli presented to the torso. The JND for stimuli applied to the torso were 30.8% and 21.9% larger on average than stimuli presented to the shoulders and arm, respectively. In the second set of experiments, we observed better discrimination for stimuli presented sequentially vs. those presented simultaneously. We also found that discrimination of stimuli presented within a single dermatome exhibited greater acuity than when stimuli were presented across dermatomes [see also (53)]. In both sets of experiments, we found systematic decreases in vibrotactile discrimination acuity with increasing age. This was a modest effect, however, in that performance in the group of older individuals in Experiment 2 improved over 3 days of testing such that their JNDs ultimately did not differ significantly from those of the younger adults.

The findings of this study are important because all tested body locations appeared to be viable stimulation sites for conveying informative vibrotactile feedback, whether sequentially or simultaneously. While people may have somewhat greater difficulty resolving stimuli of different intensities if applied to the torso, the difference in acuity across body sites appeared to be relatively modest. Although there also appeared to be a decrease in vibrotactile acuity in older adults, the magnitude of this decrement was also modest and on par with the differences in acuity observed across stimulation sites on the body. Older adults also improved their capability to discriminate vibrotactile stimuli over the course of 3 days of practice such that their final performance did not differ significantly from the two younger groups. Thus, the initial age-related decrement in vibrotactile acuity is not likely due to some hard (fixed) constraint such as a reduction in the density of peripheral receptors sensitive to vibratory stimuli. Were that so, we would not expect the initial performance deficits to resolve within just 2 or 3 days of vibrotactile stimulus intensity discrimination training.

### Factors Influencing Vibrotactile Perception

A large body of literature has identified several independent factors that influence tactile perception. Microneurographic recordings have found four groups of mechanoreceptor afferents arising from cutaneous tissues that are sensitive to vibrating stimuli (54). Pacinian corpuscles respond preferentially to vibratory stimuli in the range between 40 Hz and approximately 800 Hz, with a frequency-dependent stimulus detection curve having a maximum sensitivity near 300 Hz (48). The other mechanoreceptor groups - comprised of rapidly adapting RA afferents, slowly adapting SA I afferents, and SA II afferents (collectively called “non-Pacinian” afferents) - are generally less sensitive to vibratory stimuli over this range of frequencies [cf., (49)]; nevertheless, it is likely that the perceptual qualities of touch are determined by the combined inputs of Pacinian and non-Pacinian mechanoreceptors (48). Analysis of receptive fields finds the density of mechanoreceptors to increase in the proximo-distal direction in the hand, although this variation appears largely due to a marked increase in density of non-Pacinian receptors at the finger tips; by contrast, Pacinian receptors appear to be evenly distributed over the entire hand (54). Others have expanded the range of body sites tested with regards to the sensitivity of *vibrotactile detection*, showing that the fingertip is more sensitive in detecting sinusoidal stimuli than the hairy skin of the forearm, shoulder, and cheek [(37); see also (43)]. Additional factors influencing the detection of vibrotactile stimuli include: surface area of stimulation site and the presence or absence of a stable surround; skin temperature; body mass index; alcohol consumption; and advancing age [see (55) for a review; see also (37, 40, 42)]. Of particular relevance to the current study are the findings of Verrillo (56), who presented contours of equal sensation magnitude for vibrotactile stimuli of many different amplitudes as a function of vibration frequency. That work showed that regardless of vibrotactile stimulus displacement magnitude, the sensitivity of vibrotactile perception increases monotonically over the entire range of frequencies used in the current study. Combining Verrillo's observation with the fact that vibration intensity and frequency increase monotonically as drive current to our ERM motors is increased (Figure 1B), it follows that the perceived intensity of vibrotactile stimuli should also increase monotonically as stimulus intensity increased in our study.

Another body of literature has explored factors influencing the *discrimination* of vibrotactile stimuli of different frequencies and intensities. Early experimental work found that the skin is able to detect a difference of about 20–30% between two constant-frequency sinusoidal stimuli [see (49) for a review]. A study by Rothenberg and colleagues (57) found that the acuity of frequency discrimination is enhanced for stimuli that are frequency modulated in a way emulating the time-varying stimuli used to encode speech (57) or movement kinematics as in our pilot studies [cf., (10, 18, 19, 24)]. Interestingly, [(57)] reported that the addition of an amplitude cue to the frequency cue improved discrimination performance for each person they tested, with the average difference limen (Δ F/F) decreasing from 25% of the center frequency to 17.5% with covariation. Taken together, these findings are commensurate with the results of the current study, where we observed average difference limen Δ F/F as low as 24.2% at the shoulders in Experiment 1 (Figure 3A) and 17.9% in the C7SEQ condition of Experiment 2 (Figure 5A). More recently, Cipriani and colleagues reportedly attained an average vibrotactile difference limen of only 10% for stimuli presented to the forearm when the frequency and amplitude of stimuli varied coherently (44). Although we did not see average difference limen as low as 10% in our study, the results of Cipriani and colleagues highlight the potential benefit of selecting vibrotactile stimulators with coupled frequency and amplitude characteristics - such as those provided by ERM vibration motors - for practical sensory augmentation applications.

To the best of our knowledge, our study is the first to examine vibrotactile stimulus intensity discrimination across body locations and broad age groups using low-cost ERM actuators suitable for use in wearable sensory prostheses. Based on the literature reviewed above, we anticipated that the acuity of vibrotactile discrimination might vary not only across stimulus locations but also as a function of age. While this was indeed the case, the difference in acuity was modest both across body locations and age groups. Of the four locations tested, we found the AP torso to have the highest threshold (lowest acuity) for vibrotactile discrimination. Due to its high discrimination threshold, designers of sensory augmentation technologies may wish to avoid the AP torso as a site for applying continuously graded vibrotactile stimuli because using this location for real-time feedback could potentially decrease the accuracy of information decoding on the part of the user, potentially leading to misinterpretation or confusion. If the vibrational cues are misinterpreted, an incorrect movement will likely result. If there is confusion, the desired movement could be delayed and come too late to be useful [cf., (31)]. However, the torso location could be used for other types of feedback such as alarms [c.f., (26)], wherein discrimination between similar stimuli of different graded intensities may not be important. By contrast, the acuity of vibrotactile intensity discrimination was essentially equivalent at the arm, knee, and shoulders in Experiment 1, suggesting each site as a viable location at which to apply VTF for sensory augmentation. We also observed that across stimulation sites, the older age group had slightly higher JNDs compared to the young and middle age groups, but the young and middle groups were not different from each other. In Experiment 1, the age-dependent effect was smaller than that of the location-dependent effect, suggesting that there exists substantial latitude for individual preference in the selection of stimulation sites and/or the development of a strategy to discriminate effectively regardless of age. In Experiment 2, the older adults demonstrated marked improvement in vibrotactile acuity over just 3 days of practice, reinforcing the conclusion that age-related declines in vibrotactile perceptual acuity should not pose a limiting constraint on the use of vibrotactile sensory interfaces in that age group.

Although differences in participant discrimination performances between the sequential and simultaneous presentation of stimuli did not quite reach statistical significance in Experiment 1, our second experiment did find that simultaneously presented stimuli do have measurably higher JNDs than sequential stimuli. Those results concur with those of a prior study (33), which suggested that timing-dependent differences in acuity were due to the limitations of working memory and/or attention. Another potential cause of decreased perceptual acuity is the phenomenon of “masking,” whereby the ability to sense one stimulus is degraded or decreased by the presence of another that occurs simultaneously or very close in time (49). We speculated that one possible reason why timing-related effects did not reach statistical significance in Experiment 1 might have stemmed from the fact that approximately half of the subjects in that experiment were not naïve to vibrotactile discrimination, having had previously participated in similar sensory discrimination studies. Results of our statistical analyses in Experiment 1 showed that prior experience with the experimental procedures did not significantly improve performance, suggesting that Experiment 1 might have been somewhat underpowered to test stimulus timing effects. We therefore recruited a large cohort of naïve participants for our learning study of Experiment 2, which confirmed and extended the results of Shah et al. (33) by showing significant differences between the simultaneous and sequential presentation conditions. In any case, our current results indicate that potential impacts of factors such as masking, working memory and/or attention are likely small relative to those related to stimulus location and aging on vibrotactile discrimination. Future studies should study their potential impact on the utility of sensory augmentation systems within the context of functional tasks such as reaching and stabilizing the arm, or for improving the precision and accuracy of skilled movements such as those required in robot-assisted surgery, where cognitive factors may play an important role.

### Sensory Interfaces Reconnecting Perception to Action

The use of vibration as a mode of sensory augmentation or substitution dates back at least to the early 20th century, when Robert Gault developed a communications system that mapped audio signals from human speech into vibrotactile cues applied to the five fingers on one hand of an “observer” (58, 59). After about 25 h of practice on learning the vibrotactile cues associated with (60) words, hearing-impaired participants achieved 48% word-for-word accuracy on interpreting novel sentences, and “correct in sense” accuracy on another 28% (60). Related work continues in recent studies examining symbolic pattern discrimination in wearable vibrotactile display systems mounted to the forearm (28) or back of the hand (27).

Another line of related research seeks to encode continuous real-time information into vibrotactile stimuli to enhance control of body movements. In one example, Lieberman and colleagues provided vibrotactile feedback of joint angle error during tasks where the participant was to replicate the pose and/or motion of the upper extremity (11). The authors found a 27% improvement in replication accuracy in elbow and wrist flexion and extension motions when VTF was applied to the arm used to perform a reaching task, but no statistically significant improvement in the accuracy of rotations at the wrist and shoulder. Xu et al. (61) used “Dots” technology that could simultaneously sense postural state and provide vibrotactile feedback of standing posture. In one experiment, the Dots were placed on the torso to estimate degree of medial-lateral trunk tilt during common balance exercises, and to provide feedback when trunk tilt deviated too far from vertical. The experimenters found less trunk tilt with VTF feedback than without it.

Although the current study did not ask participants to use VTF stimuli to enhance performance of movement tasks as in our prior studies [cf., (10, 18, 19, 24, 32)], our results have identified several locations where VTF could reasonably be applied for sensory augmentation or substitution in the future. To the extent that perceptual acuity is required to successfully integrate continuous and graded-intensity vibrotactile feedback of kinesthetic information into the ongoing control of movement (18, 19), the current results support the idea that regardless of age, people may be able to discriminate stimuli provided by vibrotactile sensory augmentation technologies, and potentially use them to mitigate persistent proprioceptive deficits after neuromotor injury [cf., (10, 25)].

### Limitations and Future Directions

One limitation of this study was the unequal sample sizes across age groups; In Experiment 1, the younger age group had 16 participants, while the middle and older groups had 6 and 10 participants, respectively. In Experiment 2, the younger age group had 12 participants, while the middle and older groups had 19 and 34 participants, respectively. Despite this limitation, our study had sufficient power to identify age- and location-related differences with a medium to large effect size. With its larger sample size, Experiment 2 also found evidence for differences in the acuity of vibrotactile discrimination between the young and middle age groups as well as between sequential and simultaneous stimuli. The age-related effects we observed could arise for several reasons, including some combination of declines in peripheral somatosensory capacity and/or central cognitive ability. Because age-related effects noted on Day 1 of Experiment 2 largely disappeared by the end of Day 3 testing, it is unlikely that declines in peripheral somatosensory capacity drive the effects on Day 1 because they would not be expected to resolve over a 3 day time frame.

Another limitation arises from the fact that we only studied neurologically-intact participants in this study. Our ultimate goal is to develop sensory augmentation technologies that can substitute for proprioceptive sensations of limb posture and movement that may be lost or otherwise impaired due to neural injuries such as those cause by stroke. Some survivors of stroke have marked somatosensory deficits on one side of the body even though they retain capability to move that limb. Consequently, our initial efforts with this population have applied vibrotactile feedback to the ipsilesional, non-moving arm and hand (10, 25). The results show that some survivors of stroke can indeed use the feedback to improve control in the more involved upper extremity. The results of our current study extend those prior studies, showing that other stimulation sites besides the arm and hand exhibit comparable vibrotactile stimulus intensity discrimination capability, and may therefore be viable as stimulation sites for sensory augmentation. Such flexibility may allow future users of the technology to select stimulation sites so that the stimulation interferes as little as possible with other actions performed using the stimulated body parts. Because aging is the most robust risk factor for stroke, future study is needed to identify sources of age-related declines in vibrotactile stimulus intensity discrimination and their impact on the utility and usability of wearable sensory augmentation systems in aging populations and in individuals with neurologic injury such as survivors of stroke.

Yet another limitation of Experiment 1 is that it only sampled four different locations for VTF application. Many others are conceivable [cf., (30, 37, 43)]. While this study has shown the arm, knee, and shoulders to be viable locations for VTF application, not all possible sites are desirable in that they are either conspicuous (e.g., the head, hands) or they may interfere with important activities of independent living (e.g., tongue, hands, feet). We also did not here control for factors such as the distance between the vibration motor pairs, the relative difference in body segment mass across stimulation sites, or the spatial orientation of motor pairs at the different stimulation sites. Consequently, we cannot state unambiguously why vibrotactile intensity discrimination varied across sites, only that it did so to a modest extent. We did however design our study intentionally to reflect constraints imposed by the intended application. For example, previous work has shown that in practical applications, vibrators should be placed at least 6 cm apart to avoid mechanical cross-talk between stimulation sites [(32); see also (30)].

Finally, a critical reader might consider the use of low-cost ERM actuators to be a limitation in our study. ERM vibration motors have vibration frequency and amplitude response profiles that cannot be controlled independently (cf., Figure 1B). Although other technology options are available [cf., (62)], our ultimate goal is to reduce barriers to adoption of wearable sensory augmentation systems; the use of inexpensive ERM stimulation technology currently has a substantial cost advantage over actuators that facilitate independent stimulation of vibration frequency and intensity. Moreover, studies by Choi and Kuchenbecker (63) and Hwang et al. (64) have shown that perception of vibration intensity depends both on the frequency and amplitude of vibration [but see also (65)]. As already mentioned, experimental evidence shows that people can discriminate vibrotactile stimuli better when amplitude and frequency change coherently (44). Thus, the coupling of vibration amplitude and frequency is a beneficial feature of the low-cost ERM motors in our study. While it is possible that some individuals may find long-term use of vibrotactile stimulation to be annoying, future studies should evaluate the extent to which such subjective perceptions will limit the utility of vibrotactile stimulation in practical applications of sensory augmentation or substitution.

## Conclusions

Because vibrotactile stimulus intensity discrimination is similar in several tested locations across the body, participants could be allowed to select where they would prefer stimuli to be applied when used for sensory augmentation without compromising perceptual performance. Of the four tested sites, however, the AP torso may be a less desirable choice for encoding continuous, graded stimuli due to its lower perceptual acuity than other body locations. Furthermore, while there is a significant difference in the capability of older adults to distinguish between vibrotactile stimuli as compared to both younger and middle-aged adults, the differences were modest (with an impact smaller than that observed across stimulation sites) and they appear to resolve with just 3 days of vibrotactile discrimination training. As such, older adults may be able to benefit from sensory augmentation nearly as much as younger adults.

We expect the findings from this study will be useful for future efforts to design and implement low-cost wearable VTF systems to restore closed-loop kinesthetic feedback of the arm, legs, and/or body in those who have lost sensation. A wearable VTF system could also be useful in the case of those needing additional reminders to attend to one side of the body, for example, in patients with hemispatial neglect (66). A VTF system could also be useful for those requiring supplemental situational awareness, such as firefighters or first responders needing dynamic information of the current status of an incident or of fellow first responders (67). As these and other sensory augmentation technologies continue to be developed, knowledge of vibration discrimination capabilities across the body could increase the flexibility of location and style of the supplemental vibrotactile cues.

## Data Availability Statement

The raw data supporting the conclusions of this article will be made available by the authors, without undue reservation.

## Ethics Statement

The studies involving human participants were reviewed and approved by Marquette University Institutional Review Board. The patients/participants provided their written informed consent to participate in this study.

## Author Contributions

Conceptualization, funding acquisition, project administration, supervision, and validation: LM and RS. Data curation: EP, AT, EC, VS, and LM. Investigation: EP, AT, EC, VS, LM, and RS. Methodology and software: VS, LM, and RS. Writing: EP, EC, LM, and RS. All authors contributed to the article and approved the submitted version.

## Funding

This work was supported in part by the National Institutes of Health (Award Number R15HD093086), and by the National Science Foundation under an Individual Research and Development Plan (RAS). Any opinions, findings, conclusions, or recommendations expressed in this material are those of the authors and do not necessarily reflect the views of the NSF.

## Conflict of Interest

The authors declare that the research was conducted in the absence of any commercial or financial relationships that could be construed as a potential conflict of interest.

## Publisher's Note

All claims expressed in this article are solely those of the authors and do not necessarily represent those of their affiliated organizations, or those of the publisher, the editors and the reviewers. Any product that may be evaluated in this article, or claim that may be made by its manufacturer, is not guaranteed or endorsed by the publisher.
